# CAR-T-Cell Therapy in Multiple Myeloma: B-Cell Maturation Antigen (BCMA) and Beyond

**DOI:** 10.3390/vaccines11111721

**Published:** 2023-11-16

**Authors:** Abhinava K. Mishra, Ashna Gupta, Gunjan Dagar, Dayasagar Das, Abhijit Chakraborty, Shabirul Haque, Chandra Prakash Prasad, Archana Singh, Ajaz A. Bhat, Muzafar A. Macha, Moez Benali, Kamal S. Saini, Rebecca Ann Previs, Deepak Saini, Dwaipayan Saha, Preyangsee Dutta, Aseem Rai Bhatnagar, Mrinalini Darswal, Abhishek Shankar, Mayank Singh

**Affiliations:** 1 Molecular, Cellular and Developmental Biology Department, University of California Santa Barbara, Santa Barbara, CA 93106, USA; abhinavamishra@ucsb.edu; 2 Department of Medical Oncology (Lab), Dr. BRAIRCH, All India Institute of Medical Sciences (AIIMS), New Delhi 110029, India; aashna0506@gmail.com (A.G.); gunjandagar28@gmail.com (G.D.); researchchandra@gmail.com (C.P.P.); 3 Department of Medicine, NYU Langone Health, New York, NY 10016, USA; dayasagarbiochem@gmail.com; 4 Department of Investigational Cancer Therapeutics, The University of Texas MD Anderson Cancer Center, Houston, TX 77030, USA; aabhi.mn@gmail.com; 5 Feinstein Institute of Medical Research, Northwell Health, Manhasset, NY 11030, USA; shaque@northwell.edu; 6 Department of Biochemistry, All India Institute of Medical Sciences (AIIMS), New Delhi 110029, India; archanasingh@aiims.edu; 7 Precision Medicine in Diabetes, Obesity and Cancer Program, Department of Human Genetics, Sidra Medicine, Doha P.O. Box 26999, Qatar; abhat@sidra.org; 8 Watson-Crick Centre for Molecular Medicine, Islamic University of Science and Technology, Awantipora 192122, India; muzafar.aiiims@gmail.com; 9 Fortrea Inc., Durham, NC 27709, USA; moez.benali@fortrea.com (M.B.); kamal.saini@nhs.net (K.S.S.); 10 Addenbrooke’s Hospital, Cambridge University Hospitals NHS Foundation Trust, Cambridge CB2 0QQ, UK; 11 Labcorp Oncology, Durham, NC 27560, USA; rebeccaann.previs@labcorp.com; 12 Department of Obstetrics and Gynecology, Division of Gynecologic Oncology, Duke University Medical Center, Durham, NC 27710, USA; 13 Department of Materia Medica, State Lal Bahadur Shastri Homoeopathic Medical College, Prayagraj 211013, India; nickdeepak24@gmail.com; 14 Pratap Chandra Memorial Homoeopathic Hospital & College, Kolkata 700011, India; rik.dwaipayan@gmail.com (D.S.); preyangsee@gmail.com (P.D.); 15 Department of Radiation Oncology, Henry Ford Cancer Institute, Detroit, MI 48202, USA; abhatna1@hfhs.org; 16 Harvard T.H. Chan School of Public Health, Huntington Ave, Boston, MA 02115, USA; mrinalinidarswal@gmail.com; 17 Department of Radiation Oncology, Dr. BRAIRCH, All India Institute of Medical Sciences (AIIMS), New Delhi 110029, India

**Keywords:** CAR-T-cell therapy, multiple myeloma (MM), B-cell maturation antigen (BCMA)

## Abstract

Significant progress has been achieved in the realm of therapeutic interventions for multiple myeloma (MM), leading to transformative shifts in its clinical management. While conventional modalities such as surgery, radiotherapy, and chemotherapy have improved the clinical outcomes, the overarching challenge of effecting a comprehensive cure for patients afflicted with relapsed and refractory MM (RRMM) endures. Notably, adoptive cellular therapy, especially chimeric antigen receptor T-cell (CAR-T) therapy, has exhibited efficacy in patients with refractory or resistant B-cell malignancies and is now also being tested in patients with MM. Within this context, the B-cell maturation antigen (BCMA) has emerged as a promising candidate for CAR-T-cell antigen targeting in MM. Alternative targets include SLAMF7, CD38, CD19, the signaling lymphocyte activation molecule CS1, NKG2D, and CD138. Numerous clinical studies have demonstrated the clinical efficacy of these CAR-T-cell therapies, although longitudinal follow-up reveals some degree of antigenic escape. The widespread implementation of CAR-T-cell therapy is encumbered by several barriers, including antigenic evasion, uneven intratumoral infiltration in solid cancers, cytokine release syndrome, neurotoxicity, logistical implementation, and financial burden. This article provides an overview of CAR-T-cell therapy in MM and the utilization of BCMA as the target antigen, as well as an overview of other potential target moieties.

## 1. Introduction

Multiple myeloma (MM) results from the accumulation of abnormal plasma cells and constitutes approximately 10% of all hematological malignancies [1]. The persistence of neoplastic cells within the host arises concomitantly with the compromise of immune surveillance vis-à-vis the malignancy-associated antigen, which can lead to the progression and evasion of the malignant cells, allowing them to proliferate and establish a more aggressive tumor microenvironment. Diverse immune mechanisms contribute to the propagation of MM cells, including disruptions within B cells, T cells, and natural killer (NK) cells [2], as well as deficient antigen presentation [3] and the activation of transforming growth factor (TGF)-β, resulting in the suppression of IL-2 production and subsequent T-cell inhibition [4]. These collective events culminate in an impaired tumor-specific immune response. A deeper comprehension of MM over the past 10 years has improved therapeutic approaches. The development of immunotherapies like immunomodulatory drugs (such as pomalidomide, lenalidomide, and thalidomide), proteasome inhibitors (like bortezomib and carfilzomib), and monoclonal antibodies has significantly improved patient outcomes. These treatments focus on malignant plasma cells and suppress the immunosuppressive tumor microenvironment, which overcomes the barrier of drug resistance and ensures a lasting response [5]. Chimeric antigen receptor T-cell (CAR-T) therapy stands out as a promising immunotherapeutic strategy with encouraging results, particularly in B-cell malignancies. This success has culminated in the FDA’s approval of four CAR-T-cell therapies directed against the CD19 protein: axicabtagene ciloleucel and lisocabtagene maraleucel for diffuse large B-cell lymphoma (DLBCL), brexucabtagene autoleucel for mantle cell lymphoma, and tisagenlecleucel for acute lymphoblastic leukemia (ALL) [6,7,8].

The success of CAR T cells in CD19 B-cell malignancies has paved the way for the extension of the CAR-T approach to diverse cancer types, including MM. This article provides an overview of the utilization of CAR-T-cell therapy, with a specific focus on its diverse targets in MM. We explore existing clinical evidence and ongoing strategies to enhance the therapeutic potency and optimize the safety profile of CAR-T-cell therapy.

## 2. Components of CAR-T-Cell Therapy and the Emergence of a Different Generation of CARs

Since the 1960s, cancer immunology research has focused on a patient’s own immune cells to target tumors. Adoptive immunotherapy during the 1980s led to genetically engineered T cells, specifically CARs, which can recognize specific antigens without the need for HLA presentation. The first CAR construct was developed in 1993 through a collaborative effort led by Israeli immunologist Zelig Eshhar [9]. The process of generating CAR T cells involves isolating leukocytes from a patient’s blood through leukapheresis. Once an adequate cellular reservoir is obtained, specific T cells are induced into proliferation using cytokine interleukin 2 (IL-2) and anti-CD3 antibodies. The resulting T cells are then purified and subjected to transduction with a retroviral vector that encodes the CAR construct. This transduction gives the T cells the phenotype of CAR T cells. After this transformation, these cells are administered to patients following a regimen of lymphodepletion chemotherapy [10].

The CAR comprises three components: an extracellular domain, a transmembrane domain, and an intracellular domain. The extracellular domain is a membrane protein exposed external to the plasma membrane. In the case of CARs, the extracellular domain consists of an antigen recognition region and a hinge region [11]. The antigen recognition domain is usually a single-chain variable fragment (scFv) composed primarily of variable heavy (VH)- and light (VL)-chain regions of immunoglobins and is connected through a flexible linker [12]. The flexibility of the linker is due to the presence of hydrophilic residues from amino acids like glycine and serine. The hinge region or spacer serves as a bridge between the antigen recognition domain and the transmembrane domain. The hinge region enhances the flexibility of the scFv, reduces spatial limitations between the antigen head and CAR, and leads to the better recognition of target antigen epitopes [13]. The transmembrane domain consists of the hydrophobic alpha helix that traverses the membrane. It connects the extracellular hinge and antigen recognition domains to the intracellular signaling region, thereby anchoring the CAR to the plasma membrane, which is essential for the stability of the receptor [14].

The initiation of intracellular signaling in T cells is prompted by the engagement of antigens, which subsequently leads to the activation of the receptor’s intracellular domain, and specifically CD3ζ. This CD3ζ component serves as a pivotal element in the process of intracellular signaling within T cells. Upon the recognition of antigens, the receptors aggregate, culminating in the generation of a signaling cue that subsequently propagates to the T cell. After this event, the commencement of intracellular activation within T-cell signaling ensues. This activation permits the recruitment and phosphorylation of immunoreceptor tyrosine-based activation motifs (ITAMs), which are localized in the cytoplasmic domain of CD3ζ [14].

**Figure 1:**
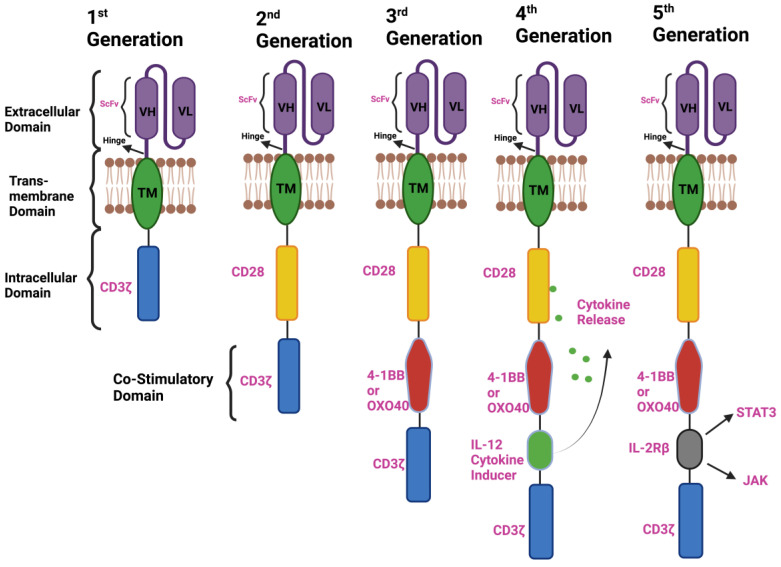
**Basic structures and generations of CAR T cells: First-generation CARs** have only one signal structure domain (CD3ζ) and no co-stimulatory domain and are the initiators of T-cell receptor intracellular signaling. **Second-generation CARs** are engineered to contain one co-stimulatory domain to enhance cytotoxicity and persistence. **Third-generation CARs** contain two co-stimulatory domains: CD28 4-1BB or OXO40. **Fourth-generation CARs**, which are also called TRUCKs, possess a cytokine-induced domain IL-12 (cytokine inducer). **Fifth-generation CARs** have an additional intracellular domain, creating a truncated cytoplasmic IL-2 receptor β-chain domain with a binding site for transcription factor STAT3.

To address the requirement for enhanced T-cell activation beyond the CD3ζ co-stimulatory domain, the incorporation of various co-stimulatory moieties (e.g., CD27, CD28, CD134 (OX40)) within the extracellular domain of the CAR has been undertaken. The integration of these supplementary domains has given rise to distinct generations of CARs. These iterations are tailored to accommodate the diverse challenges posed by the variable tumor microenvironment, prevalent in both hematological and solid malignancies (Figure 1).

### 2.1. First-Generation CAR T Cells

Initially, first-generation CARs were composed of two distinct molecules: the T-cell receptor (TCR) α and β chains, each fused to the light and heavy chains of an antibody variable region. These CAR components were later integrated into a single molecule, with the intracellular domains of T-cell co-receptors and the extracellular domains of ITAM-containing immunoreceptors, the Fcϒ chain or CD3ζ chain, fused to the transmembrane [15]. In order to preserve the single-chain architecture through the integration of both heavy- and light-chain antibody fragments, a linker was employed to covalently connect these chains, resulting in the formation of a single-chain variable fragment (scFv). To construct the pioneering single-molecule chimeric antigen receptor (CAR) aimed at targeting cancer cells adorned with specific antigens, this scFv was subsequently conjugated with either the ζ or γ chain (Figure 1) [16]. Eshhar and his team engineered two functional “first generation” CARs targeting antigens native to tumor cells: the alpha folate receptor and HER2 (ERBB2). While these initial CAR T cells efficiently killed tumor cells in laboratory settings, replicating these results in animal models proved challenging [16].

Jensen and colleagues developed the first CAR-T-cell therapy against CD19, a B-cell marker expressed in most leukemia cells. These human anti-CD19 CAR T cells effectively killed CD19-expressing leukemia cells in vitro [17]. Although many studies showed promise in vitro, they did not translate into effective tumor clearance in in vivo settings [8,18,19]. While the CAR T cells had a good safety profile, their clinical effectiveness was not achieved due to their insufficient capacity to produce enough interleukin-2 (IL-2).

### 2.2. Second-Generation CAR T Cells

Second-generation CAR T cells have overcome limitations associated with first-generation CARs, and almost all CARs approved for clinical use are second-generation. To achieve the optimal level of T-cell activation, the major challenge was to achieve co-stimulation for physiological downstream CAR signaling. The immunoglobulin superfamily member CD28 initially co-stimulated previously unstimulated T cells. When CD28 was coiled to either CD80 or CD8, their ligands, T-cell activation did not take place. However, robust T-cell activation occurred when both the T-cell receptor (TCR) and CD28 ligands were bound simultaneously. This cooperative phenomenon was termed “co-stimulation.” [20].

To integrate co-stimulation into chimeric antigen receptors (CARs), researchers initially combined CD28 with activation signals from an ITAM-containing receptor. This was achieved through two distinct approaches: one involved a single-chain variable fragment (scFv) linked to the intracellular and transmembrane domains of CD28, while the other utilized the co-expression of this construct with a CAR containing a ζ chain. The original single-molecule design contained CD28, a ζ chain, and a single scFv, targeting CD33 expressed on AML cells. This construct demonstrated a notable increase in interleukin-2 (IL-2) secretion compared to a CAR containing only the ζ chain [21]. Although the enhancement was relatively modest, it laid the foundation for developing more robustly co-stimulated “second-generation” CARs that exhibit in vivo activity. Most research groups focused on creating CARs that target antigens found in solid tumors [22]. In these designs, the CD28 co-stimulatory domain was extensively utilized in second-generation CARs [23]. Two independent research groups chose to replace CD28 with 4-1BB, a member of the TNFRSF, in their anti-CD19 CAR constructs [24]. To curb excessive T-cell activation, the co-stimulatory role of CD28 can be substituted by CD137 in T cells. This replacement has been observed to play a pivotal role in the development of cytotoxic T-cell memory. Dario Campana and his research team showed that the co-stimulation of the CD137 cytoplasmic domains in CD19-specific CAR constructs improved the cytotoxicity against leukemia cells compared to CD19-specific CARs without the CD137 domain [24]. The choice of the co-stimulatory domain has been linked to the differences in activity and persistence of the CAR T cells, and studies that are focused on characterizing the effects of various co-stimulatory domains are ongoing beyond CD28 and 4-1BB. New domains like OX-40, ICOS, and CD27 are of particular interest and are actively being pursued in clinical trials.

### 2.3. Third-Generation CAR T Cells

Third-generation CARs, which typically include two co-stimulatory domains, CD28 and 4-1BB, alongside the CD3ζ activation domain, significantly enhance the antitumor efficacy when human CAR-transduced T lymphocytes are adoptively administered to mice-bearing tumors [25]. Third-generation CAR-Ts exhibit higher levels of phosphorylation of their downstream targets than second-generation CAR-Ts, resulting in enhanced intracellular signaling [1,2]. This allows the third-generation CAR-Ts to support better growth, differentiation, and expansion into memory T-cell subsets [1,3]. In CAR-T-cell therapy for B-cell malignancies, third-generation CAR T cells may increase the risk of infection due to heightened B-cell aplasia. Third-generation CAR T cells are also associated with an elevated potential for immune-related adverse effects and severe instances of cytokine storms. Despite this, their favorable attributes warrant their potential adoption as successors to the preceding CAR generations [26].

### 2.4. Fourth-Generation CAR T Cells

A fourth generation of CAR-transduced T cells, referred to as T cells redirected for universal cytokine killing (TRUCKs), was engineered with the primary aim of expanding the applicability of CARs to target a wide range of cancers, including both solid tumors and hematological malignancies (Figure 1). These “armored CAR” T lymphocytes are genetically engineered to release specific transgenic factors upon the activation of their CARs through the targeted antigen. This genetic modification typically induces the secretion of pro-inflammatory cytokines, like IL-12 [26]. As a result, the T-cell proliferation and function are further enhanced, and, most importantly, there is an increased recruitment of innate immune effectors to the tumor microenvironment. This results in improved tumor killing while mitigating cytokine-mediated systemic toxicity [27]. The increased efficacy and promise of this strategy in targeting solid tumors have been highlighted by preclinical studies of IL-12-secreting CAR-transduced T cells directed against CD19 and VEGF receptor-2 (VEGFR-2), as well as subsequent studies of armored CAR T cells with alternative payloads, like 4-1BBL and CD40L.

### 2.5. Next or Fifth-Generation CAR T Cells

CAR-T-cell treatment has undergone significant development to improve its effectiveness, safety, persistence, and proliferation. The fifth or future generation of CARs is being developed in this context [13], and they have an additional intracellular domain that fine-tunes the activity of these CARs with modular switches (Figure 1). The CARs comprise truncated intracellular domains of cytokine receptors, such as the IL-2R chain, that have a motif for binding transcription factors like STAT-3/5. The most promising strategy involves the insertion of IL-2 receptors, which enables JAK/STAT-pathway activation in an antigen-dependent manner [28]. The successful incorporation of a drug-dependent OFF switch leading to CAR depletion or an ON switch leading to activation has recently been reported.

## 3. Emergence of B-Cell Maturation Antigen (BCMA) as a Promising Target for CAR-T-Cell Therapy in Multiple Myeloma

The TNFRSF17 gene encodes the B-cell maturation antigen (BCMA) (a member of the tumor necrosis factor (TNF) receptor family), and it is situated on chromosome 16, spanning approximately 2.92 kilobases comprising three exons and two introns. The BCMA is primarily responsible for the regulation of B-cell survival, proliferation, and maturation. It collaborates with other members of the TNFR superfamily, including B-cell activating factor (BAFF-R) and TACI, known as the transmembrane activator and calcium modulator and cyclophilin ligand interactor. The human BCMA has four splice variants, each distinguished by specific features, like receptor-binding affinities, intracellular-domain-signaling capabilities, and membrane-anchoring characteristics. The BCMA is a particularly attractive target due to its apparent selectivity of expression. The BCMA is predominantly expressed on plasma cells and plasmablasts but is not expressed on memory or naïve B cells and is absent from non-hemopoietic tissues [29]. The BCMA is expressed in most patients with MM but sometimes its expression is low. Studies have shown that the BCMA expression is constricted to the mature B-lymphoid compartment, making it a promising target in patients with MM [30]. Many studies have reported the expression of BCMA on MM cells, and selectively on plasma cells in normal tissues [23,31,32]. Clinical samples have also been analyzed to explore the range of BCMA expressions [33,34].

### 3.1. Structure and Interaction of BCMA

**Figure 2:**
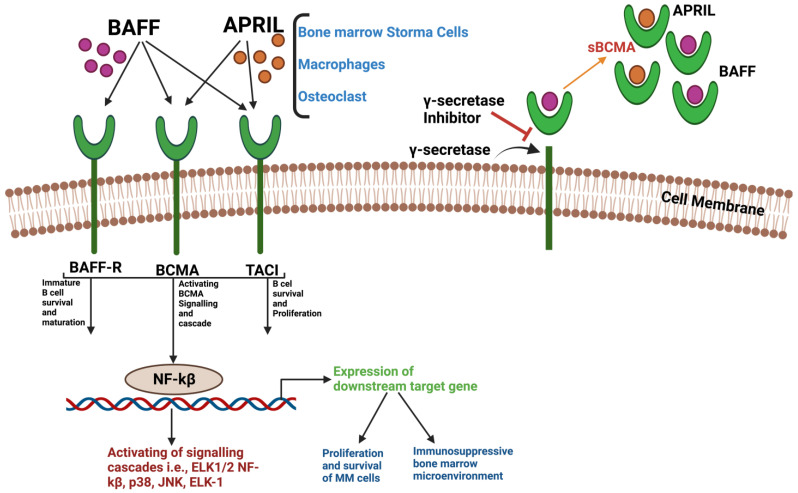
**Significance of BCMA signaling pathway in plasma cells:** BAFF and APRIL are two natural ligands for BCMA. APRIL can bind with both TACI and BCMA and has a higher binding affinity with BCMA than BAFF. BAFF is a ligand for BAFF-R and can also bind with BCMA. The binding of APRIL and BAFF with BCMA results in the activation of different pathways in MM cells, like AKT, P38, NF-kβ, and JNK. γ-secretase can cleave the BCMA membrane and release sBCMA, which can also bind with BAFF and APRIL.

TNF superfamily members comprise trimeric proteins that are characterized by their structures as either membrane-bound or soluble ligands, engaging specific receptors. A sequence alignment conducted between murine and human BCMA proteins revealed a conserved motif featuring six cysteines located at the extracellular terminus. This conserved motif suggests the BCMA’s membership within the TNFR family. Notably, the BCMA is positioned within the TNFR subfamily, which encompasses APRIL (CD256, TNFSF13) and B-cell activating factor (BAFF) (also recognized as BLyS, TALL-1, CD257, or TNFSF13B). These ligands interact with three distinct cell-surface receptors of the TNFR family: TACI (CD267, TNFRSF13B), BAFF-R (also acknowledged as BR3, CD268, or TNFRSF17), and BCMA (CD269, TNFRSF13C) [29]. The proliferation-inducing ligands APRIL and BAFF are the two ligands for the BCMA (Figure 2). BAFF is primarily produced by stromal cells and macrophages in the bone marrow in a paracrine way. The BCMA, BAFF, and TACI all contain a single cysteine-rich domain (CRD). APRIL binds with a significantly higher affinity than BAFF with BCMA, and it also binds with TACI. Upon the binding of APRIL or BAFF to the BCMA, multiple survival and growth signaling cascades are activated in MM cells via the activation of different pathways, like AKT, P38 KINASE, NF-kβ, and Elk-1 [30] (Figure 2). In MM cells, the binding of BCMA to APRIL or the overexpression of the BCMA promotes the proliferation and survival of MM cells via the activation of different cascades, like the AKT, ERK1/2, and NF-kβ pathways. By modulating cell cycle checkpoints, most frequently through NF-kβ, the activation of these pathways stimulates cell proliferation. This results in the production of cell adhesion molecules, like VEGF and IL-8, and the upregulation of anti-apoptotic proteins, like BCL-2 and BCL-XL, as well as immunosuppressive molecules like PD-L1 and TGF-β. These factors lead to the increased survival of MM cells [35]. The BCMA membrane can be split by γ-secretase, which then releases the soluble BCMA (sBCMA) into the plasma (Figure 2). CAR-T-cell therapy targeting the BCMA has been developed over the past decade, harnessing the advantages of both molecular antibody-like target specificity and the cytotoxicity of T cells. CAR-T-cell therapies in MM have shown impressive clinical results [36,37]. Some of them are already in use and use BCMA as a target antigen, while others that target alternate antigens are in clinical trials, as listed in Table 1.

### 3.2. Idecabtagene Vicleucel (Ide-Cel, bb2121)

Idecabtagene vicleucel, also known as Ide-cel and bb2121, is a second-generation, FDA-approved CAR-T-cell therapy targeting the BCMA. Ide-cel consists of autologous T cells and is transduced with a lentiviral vector expressing BCMA CAR, and it was approved in 2021. The composition of this therapeutic product comprises a murine anti-BCMA single-chain variable fragment, incorporating a 4-1BB co-stimulatory domain, a CD3ζ signaling domain, a CD8α hinge, and a transmembrane domain. Comprehensive preclinical investigations have demonstrated the potent in vitro cytotoxicity of Ide-cel against multiple myeloma (MM) cells. Remarkably, this cytotoxicity is independent of the BCMA expression levels or the soluble form of BCMA (sBCMA) [37]. Notably, Ide-cel exhibited rapid and sustained tumor eradication in an in vitro study involving patients with relapsed and refractory MM (RRMM). Building on these promising outcomes, a phase 1 clinical trial (CRB-401, NCT02658929) was conducted in RRMM patients who had previously undergone three or more therapeutic interventions and were refractory to treatment. The results of the trial underscore the effectiveness of Ide-cel in this patient cohort, characterized by an impressive overall response rate (ORR) of 73% and a complete response (CR) rate of 33%. Patients in the trial received Ide-cel doses of 50, 150, 450, or 800 × 10^6^ cells. Out of 72 patients, 33 consecutive patients with a median of 11.3 months of follow-up were reported. Cytokine release syndrome (CRS) was evident in 76% of all patients, which was of grades 1 and 2 [38]. Given the safety and efficacy of the study, a phase 2 clinical trial of bb2121 (KarMMa, NCT03361748) enrolled patients who had received three lines of therapies, including IMiD and proteasome inhibitors (PIs). A dose of 150–450 × 10^6^ cells was given to the patients with RRMM. A total of 128 patients received infusions, of which 4 patients received 150 × 10^6^ cells, 70 received 300 × 10^6^ cells, and 54 patients were given a dose of 450 × 10^6^ cells. Follow-up was after 11.3 months, and 94 patients showed clinical responses. Patients who received 450 × 10^6^ cells achieved an 81.5% ORR and a 35.2% CR, indicating that a higher dose of CAR T cells might be associated with a better response [39].

### 3.3. Ciltacabtagene Autoleucel

Cilta-cel is a genetically engineered CAR-T-cell therapy targeting the BCMA. It falls under the second-generation CARs, equipped with two OBAMA-binding scFv domains, a transmembrane component, a CD3ζ signaling domain, and a 4-1BB co-stimulatory domain. This process involves the modification of the patient’s T cells with a transgene encoding the CAR, enabling the identification and subsequent elimination of tumor cells expressing the BCMA. Cilta-cel is currently indicated for the treatment of RRMM after Idecabtagene vicleucel (bb2121) CAR-T-cell therapy. Cilta-cel is a second approved immunotherapy for patients with advanced MM. The FDA granted approval for cilta-cel to be used in adult patients with MM on 28 February 2022. This decision was based on the findings from the CARTITUDE-1 study (NCT03548207), which included both phase 1b and phase 2 open-label trials. These trials involved individuals who had undergone at least three prior lines of therapies. Out of the 113 participants that were enrolled, 97 received a single cilta-cel infusion that targeted a dose of 0.75 × 10^6^ CAR T cells per kilogram (within the range of 0.5–1.0 × 10^6^). The median follow-up period extended to 12.4 months, and the observed overall survival (OS) and progression-free survival (PFS) rates were 89% and 77%, respectively, at the 12-month mark. Among the 97 patients, 73 underwent bridging therapy, primarily utilizing various combinations of bortezomib, pomalidomide, carfilzomib, corticosteroids, and daratumumab. It is notable that 49% of patients experienced an increase in the tumor burden, with only 45% exhibiting a response to the bridging therapy. Currently, clinical trials are being conducted for the CARTITUDE-2 phase 2 study to assess the efficacy of cilta-cel [40]. The cumulative results from CARTITUDE-1 and CARTITUDE-2 underscore the potential of cilta-cel as a promising therapeutic option for patients with RRMM. The ongoing CARTITUDE-4 study (NCT04181827) continues to expand our understanding of cilta-cel’s utility.

### 3.4. JCARH125 (Orva-Cel)

Orva-cel is another BCMA CAR-T-cell product transduced with a genetically modified lentiviral vector to express a BCMA CAR construct. The construct consists of unique BCMA-specific, human single-chain variable fragments, co-stimulatory domains (4-1BB), and CD3ζ signaling domains. A phase 1/2 trial (EVOLVE trial) (NCT03430011) was conducted on 51 patients with RRMM who had received orva-cel at 300, 450, and 600 × 10^6^ CAR T cells after lymphodepletion with cyclophosphamide and fludarabine. The efficacy data showed an ORR of 91%. However, two patients experienced dose-limiting toxicities: one had a grade 3 neurological event after the infusion of 300 × 10^6^ CAR T cells, and another had grade 4 neutropenia after the infusion of 300 × 10^6^ CAR T cells. Additionally, 14% of patients exhibited grade 3 or higher infections. However, cytokine release syndrome (CRS) was manageable, with 78% of cases responding to tocilizumab and/or steroids and 14% to anakinra, while 6% required vasopressors. The study found that orva-cel at doses of 300, 450, and 600 × 10^6^ CAR T cells in individuals with RRMM showed promising efficacy [41].

### 3.5. JNJ-4528 (LCAR-B38M)

JNJ-68284528, alternatively recognized as JNJ-4528, represents a second-generation bispecific chimeric antigen receptor (CAR) T-cell therapy designed to target the B-cell maturation antigen (BCMA) specifically. It has two distinct epitopes and co-stimulatory domains, which improves the binding avidity to BCMA-expressing cells. A single-arm, multicenter, open-label phase 1 and 2 study in patients with RRMM was initiated in China to assess its efficacy and safety. The first-in-human study (NCT03090659) in 74 patients with RRMM showed durable responses with a manageable safety profile [42].

In December 2017, Legend and Janssen jointly introduced LCAR-B38M/JNJ-4528, a commercialized CAR-T-cell therapy. The therapy comprised three infusions delivered over 7 days, with a median dose of 0.5 × 10^6^ cells/kg, adjusted based on weight. Lymphodepletion was facilitated using CTX at 300 mg/m^2^ administered three times. The regimen was administered to 57 eligible patients with a median age of 54 years who had previously experienced treatment failures and received bortezomib. Follow-up assessments were conducted for 19 months. The phase 1b/2 clinical investigation termed CARTITUDE-1 (NCT03548207), aimed at evaluating LCAR-B38M/JNJ-4528, expanded its reach to encompass Europe. Concurrently, a phase 2 clinical study, designated CARTIFAN-1 (NCT03758417), was initiated in China to further explore the therapeutic potential. The safety profile exhibited by LCAR-B38M is akin to those of prior BCMA-targeted CAR-T-cell therapies. Notably, preliminary findings from this exploratory analysis suggest that LCAR-B38M holds significant promise as an efficacious intervention for patients with RRMM [43]. Particularly notable are the profound and sustained therapeutic effects, particularly following lymphodepletion using Cy/Flu (cyclophosphamide/fludarabine). These outcomes imply that variations in the long-term efficacy may be influenced by diverse lymphodepletion regimens, albeit the limited sample size precludes definitive conclusions, and other contributing factors remain plausible [35]. The ongoing study continues to monitor the follow-up and long-term safety.

Presently, a phase 2 confirmatory study (CARTIFAN-1, NCT03758417, LCAR-B38M) is underway in China, while a phase 1b/2 clinical trial (CARTITUDE-1, NCT03548207, JNJ-4528) is enrolling in the United States.

## 4. Non-BCMA CAR-T-Cell Targets

CAR-T-cell therapies targeting the BCMA have demonstrated promising results in patients with RRMM. However, the long-term follow-up of these patients has shown that some of them experience BCMA-negative relapses, like the case of CD19-negative relapses in patients undergoing CAR-T-cell therapies targeting CD19 in other forms of leukemia. This has led to the development of alternative CAR-T targets in MM.

These new targets can be used as mono-targets or for the development of bispecific CARs with the BCMA to achieve a more durable response. Several promising non-BCMA CAR-T targets in MM have emerged, including CD138 (syndecan-1), which is abundantly expressed on myeloma cells [44]; GPRC5D (G-protein-coupled receptor, class C, group 5, member D), a receptor implicated in cell proliferation and found on myeloma cells [45]; and FcRH5 (Fc receptor homolog 5), a receptor linked to the immune response present on myeloma cells [46]. While these targets are currently in the investigative phase, their potential to introduce novel therapeutic options for patients with RRMM is significant.

### 4.1. GPRC5D

G-protein-coupled receptor, class C, group 5, member D (GPRC5D) is a human protein encoded by the *GPRC5D* gene. GPRC5D is a surface receptor that is expressed on various bone marrow plasma cells and myeloma cell lines from patients with MM. The expression of GPRC5D is minimal on bone marrow samples of diffuse large B-cell lymphoma and acute leukemia, and, in normal tissue, it is limited to the skin (eccrine glands and hair follicles). This makes GPRC5D an attractive target for the development of novel therapies for patients with MM [47].

Preliminary preclinical investigations have revealed that GPRC5D CAR T cells exhibit substantial efficacy in the MM xenograft model. Additionally, it is noteworthy that in in vivo models, GPRC5D CAR T cells do not elicit alopecia or manifest any other clinical indications of skin damage [45,48]. MCARH109, a CAR-T-cell treatment that targets GPRC5D in patients with extensively pretreated RRMM, was tested in a phase 1 human dose-escalation study. Four doses of MCARH109 were given to 17 patients, including those who had relapsed after receiving BCMA CAR-T-cell treatment. In the cohort, 71% achieved partial responses. In comparison, 55% of patients who received 25 × 10^6^, 50 × 10^6^, and 150 × 10^6^ CAR T cells achieved excellent partial responses or better. A total of 88% (*n* = 12) of patients showed grade 1 or 2 CRS. In contrast, one patient who had received 450 × 10^6^ cells showed grade 4 CRS associated with ICANS. Two other patients receiving the same dose (450 × 10^6^ cells) developed grade 3 cerebellar disorder [48]. Another phase 1 study conducted by Bal et al. evaluated the efficacy and safety profile of BMS-986393 (CC 95266), a GPRC5D-targeted CAR-T-cell therapy, in heavily pretreated patients with RRMM. Out of 19 patients, 17 went into remission, comprising a 100% overall survival rate (*n* = 10) for those who had not received anti-BCMA therapy before and a 78% overall survival rate (*n* = 9) for those who had progressed after receiving anti-BCMA therapy. However, no reports of grade 3 CRS, ICANS, or on-target/off-tumor activity were reported (NCT04674813) [49]. The single-arm phase 2 trial evaluating anti-GPRC5D CAR-T-cell therapy in patients with RRMM showed notable results. The trial included individuals who had been exposed to anti-B-cell maturation antigen (BCMA) CAR-T-cell therapy previously. The overall response rate reached 91%, with all nine patients who had previously undergone anti-BCMA CAR-T-cell therapy achieving partial responses or better. Cytokine release syndrome manifested in 76% of patients, primarily at grade 1 or 2, and neurotoxicity was detected in three individuals. These observations collectively indicate that anti-GPRC5D CAR T cells show potential as a viable treatment approach for multiple myeloma patients who have faced disease progression after prior anti-BCMA CAR-T-cell therapy [50].

Another phase 1 study, “POLARIS”, was conducted by a Chinese group. This CAR-T-cell therapy targets GPRC5D ((OriCAR-017) NCT05016778). Nine patients received OriCAR-017 CAR T cells in a dose-escalation phase (three patients received 1 × 10^6^ CAR T cells/kg, three received 3 × 10^6^ CAR T cells/kg, and three received 6 × 10^6^ CAR T cells/kg). An overall response rate of 100% was achieved. However, no mortality was seen. Grade 3 toxicity was observed, including thrombocytopenia, leukopenia, neutropenia, and anemia [51].

In addition to GPRC5D CAR-T-cell monotherapy, BCMA and GPRC5D dual-target CAR-T-cell therapies are being studied in clinical settings in patients with RRMM (NCT05325801, NCT05509530) [52,53]. Another clinical trial (NCT05431608) is ongoing in which the concurrent administration of BCMA-targeted CAR T cells and GPRC5D-targeted CAR T cells is being studied in clinical settings for RRMM patients [54].

### 4.2. SLAMF7/CS1

The SLAMF7 (CD319, CS-1) receptor belongs to the signaling lymphocytic activation molecule (SLAM) receptor family [55]. SLAMF7 functions as a homotypic receptor and, after activation, its cytoplasmic domain recruits different SH2 domain-containing proteins. In doing so, SLAMF7 is capable of modulating a variety of immune cell-specific functions across many immune cell types.

The introduction of elotuzumab, the first SLAMF7-specific humanized monoclonal antibody, was the first FDA-approved antibody for the treatment of MM. Elotuzumab in combination with pomalidomide or lenalidomide has shown clinical efficacy in relapsed MM patients [56,57]. A preclinical study by Gogishvili et al. on anti-SLAMF7 CAR T cells derived from elotuzumab demonstrated rapid cytolysis in relapsed or refractory myeloma cells in vitro. Further, they showed the elimination of extramedullary myeloma cells in the xenograft model. However, due to the presence of SLAMF7 on normal B cells and T cells, these CAR T cells demonstrated the induced selective fratricide of SLAMF7+/high T cells, B cells, and NK cells, while leaving the SLAM7/low fraction in each subset. This could represent a challenge in the clinical application of such CAR T cells [58]. Despite the promising target for MM in many preclinical studies [59,60], anti-SLAMF7 CAR-Ts may increase the risk of infections due to the elimination of SLAMF7-expressing leukocytes, like dendritic cells and NK cells. To avoid these side effects, a “suicide gene” approach has emerged as a viable option to kill anti-SLAMF7 CAR T cells. A SLAMF7 CAR T cell incorporated with the suicide gene was developed for phase 1 clinical trials for enhanced safety and tolerability (NCT03958656). Amatya et al. also developed an anti-SLAMF7 CAR expressing an anti-SLAMF7 antibody and a suicide gene, inducible caspase 9 (IC9), in genetically engineered T cells. This novel anti-SLAMF7 CAR specifically identifies SLAMF7-expressing target cells and eliminates SLAMF7 expression from mice [60].

Employing allogeneic CAR-Ts has been a significant step forward in bringing down the cost of CAR-T-cell therapy and its timely availability. Transplanted donor cells frequently destroy the patient’s normal cells, a side effect known as graft-versus-host disease (GVHD) [61]. To avoid rejection, researchers developed a UCARTCS1, an allogenic anti-SLAMF7 CAR T cell. It is the first “off the shelf” allogeneic CAR T cell for MM and has gained FDA approval for clinical trials (MELANI-01). UCARTCS1 was produced by employing healthy allogeneic T cells and TALEN gene-editing technology. This process aimed to eliminate the expressions of endogenous TCR and SLAMF7, thereby reducing the likelihood of GVHD and T-cell fratricide. The product disrupts the T-Cell Receptor Alpha Constant (TRAC) gene of the T-Cell Receptor alpha-beta (TCRab) receptor and CS1 gene. These modifications minimize the risk potential of GVHD and avoid T-cell fratricide [62]. To increase the durability of T cells and to overcome the problem of antigen loss, Zah et al. designed and optimized BCMA/CS1 CAR T cells. These are called “tandem” CARs or bispecific CARs. The scFv of these CARs contains two ligand binding sites (one binds with SLAMF7 and the other with BCMA) connected in tandem. This study found that BCMA/CS1 CAR T cells are more effective than the individual activity of T cells expressing CARs. Furthermore, combination therapy with anti-PD-1 antibody shows increased tumor clearance in vivo [63]. The European team also developed a unique SLAMF7 CAR-T-cell model, “CARAMBA”, for virus-free CAR gene transfer by utilizing Sleeping Beauty transposon technology. This strategy can potentially accelerate vector procurement and CAR-T manufacturing and provide an effective, safe, and economically viable treatment. The CARAMBA clinical trial (Phase 1/2; EudraCT No. 2019-001264-30/CARAMBA-1) is evaluating the safety, feasibility, and anti-myeloma efficacy of autologous SLAMF7 CAR T cells [64].

### 4.3. CD38

Cluster of differentiation 38 (CD38) is a multifunctional transmembrane ectoenzyme with cyclase and NADase activity. CD38 is expressed in various immune cells, such as plasma cells and lymphocytes. The aberrant expression of CD38 in a range of tumor types is linked to carcinogenesis [65]. The high expression of CD38 on MM cells appears to be a promising target for antibody treatment. The success of various anti-38 monoclonal antibodies, such as daratumumab and isatuximab, has opened the door for the development of anti-38 CAR T cells [65,66].

Preclinical investigations demonstrating anti-CD38 CAR-T-cell therapy’s anti-myeloma potential have catalyzed numerous clinical trial commencements [67,68]. An open-label phase 1 clinical trial has been initiated to explore the therapeutic efficacy of CAR2 anti-CD38 A2 CAR T cells when used as a monotherapy for the management of relapsed and refractory multiple myeloma patients (NCT03464916) [69]. Moreover, to improve the CAR specificity and counteract antigen escape, different studies have been conducted to develop tandem CARs. Yaru et al. developed a novel BCMA-OR-CD38 tandem CAR T cell that can trigger robust cytotoxicity to target cells compared to T cells expressing the BCMA or CD3. Furthermore, they assessed this study in vivo and found that after the infusion of BCMA-OR-CD38 Tan CAR T cells, a complete tumor clearance was achieved [70].

A single-arm preclinical study assessed the safety and efficacy of BCMA-CD38 bispecific CAR T cells in 16 patients with RRMM. They found that BCMA-CD38 CAR T cells have a better killing effect on target cells in vitro than CD38 CAR-Ts or BCMA CAR T cells. Next, they found that patients receiving BCMA-CD38 bispecific CAR T cells have a low recurrence rate of disease and manageable CRS, which shows that BCMA-CD38 bispecific CAR T cells could be a promising treatment strategy for RRMM [71]. A similar study was conducted that showed that a combination of murine anti-CD38 CAR T cells with humanized anti-BCMA CAR T cells constitutes an effective treatment approach with long-term responses in patients with RRMM (ChiCTR1800017051) [72]. As CD38 is also expressed in NK cells, activated T cells, and muscle cells, there is a chance of developing “on-target, off-tumor” toxicity. To reduce the toxicity, research focuses on developing affinity-optimized CD38 CAR T cells that mostly target plasma cells with high CD38 expression while sparing normal cells with low CD38 expression [73].

### 4.4. CD19

Cluster of Differentiation 19 (CD19) is a transmembrane protein encoded by the CD19 gene. In humans, CD19 is expressed in all lineages of B cells. A minor subset of myeloma cells also express CD19; however, their expression is low. Studies have shown that the expression of CD19 in myeloma stem cells is associated with drug resistance and relapse-promoting properties that are responsible for the fatal nature of MM. This finding was the rationale for a clinical trial using a CAR against CD19 [74]. The administration of CTL019, an anti-CD19 CAR T cell, to patients who have received melphalan and autologous stem cell transplantation (ASCT), a standard therapy for MM, aims to further enhance the treatment outcomes and potentially improve disease control. Out of ten patients, six patients achieved very good partial responses after receiving ASCT + CTL019, two patients achieved partial responses, and two patients experienced progressive disease. Overall, the product was well tolerated and only minor side effects were seen (NCT02135406) [75].

To further improve the efficacy and reduce relapse, Wang et al. designed a bispecific CAR-T targeting CD19 and BCMA to assess the overall response, long-term outcomes, and safety. In a single-arm phase 2 trial conducted in patients with RRMM, the overall response rate of patients was 92% (57/62) [76].

Shi et al. tested the efficacy and safety of sequential anti-CD19 and anti-BCMA CAR-T-cell and lenalidomide maintenance after ASCT. The ORR in patients (*n* = 10) was 100% (10/10), while the CR rate was 10% (1/10). At the median follow-up of 42 months, 70% of patients (7/10) showed minimal residual disease negativity for more than 24 months [77]. Further investigation is warranted to optimize the CAR-T-cell dosages for this therapy along with the combination with other antigens, like BCMA, to develop more durable CARs.

### 4.5. CD138

CD138, also called syndecan-1, is a surface protein found on the surfaces of plasma and myeloma cells. CD138 is important for accumulating survival signals and for cell adhesion. The high expression of CD138 on MM cells makes it a desirable target. Gourd et al. showed that the CD138 antibody, when combined with radioimmunotherapy, improved the long-term survival in vivo [78]. A group tested a second-generation CD138-directed 4-1BB/CD3 CAR construct as monotherapy for patients with RRMM. Five patients were infused with CART-138 cells after receiving a combination of induction therapy. Out of the five patients, four patients had stable disease for more than three months. However, one patient experienced disease progression, despite a temporary reduction in myeloma cells, and had detectable CART-138 in the bone marrow for 3 months. The data obtained were from a small sample size; hence, the results might not have been enough to be meaningfully interpreted (NCT01886976) [79]. Ongoing research includes a phase 1 study targeting CD138 with autologous CD138-CART cells for RRMM patients (NCT03672318) [80].

Despite being an attractive target for MM, CD138 may also have limitations. While overexpressed in myeloma cells, CD138 is also present in other normal tissues, like epithelial cells. This highlights the possibility of on-target/off-tumor effects, which was evident in previous clinical trials evaluating an anti-CD138-antibody–drug conjugate in the treatment of MM, as some patients experienced severe mucosal and skin toxicity. One study has evaluated the efficacy and safety of CD138 CAR preclinically. T cells from both healthy donors and multiple myeloma (MM) patients were genetically modified with a CD138 CAR, and they exhibited anti-myeloma activity. Future research should concentrate on the MM microenvironment, which is rich in cytokines and immunosuppressive cells that may restrict the antitumor action of CAR-Ts, in order to prevent antigen escape [44].

### 4.6. NKG2D

NKG2D is an activating receptor expressed by natural killer cells and effector T cells. The NKG2D receptor recognizes and binds to diverse ligands, such as MHC class I chain-related molecules A and B (MICA, MICB) and UL16-binding proteins (ULBP1–6). These ligands are often induced by a viral infection or by tumorigenic insult; however, they are not expressed on healthy tissue. More than 70% of human cancers have upregulated NKG2D ligand expression, including MM [81].

Sentman et al. showed that NKG2D CAR T cells effectively cure established MM, ovarian cancers, and lymphoma in vivo [82]. Additionally, it has been demonstrated that NKG2D CAR T cells inhibit the growth of tumors that express different NKG2D ligands. However, like CAR toxicity, NKG2D CAR toxicity is associated with the release of inflammatory cytokines. Therefore, Baumeister et al. conducted a phase 1 dose-escalation study to evaluate the feasibility and safety of NKG2D chimeric antigen receptor (CAR) T cells in a small cohort of five patients with MM. No CRS, CAR-T-cell-related neurotoxicity, or significant autoimmune reactions were observed, which supports the further development of this approach [83].

The development of autologous NK cells (NKAE) showed promising results without inducing toxicity, thereby establishing a platform for the development of autologous CAR-NKAE. CAR-NKAE cells exhibit increased cytotoxicity against MM cells both in vitro and in vivo, demonstrating that they could be a promising strategy against MM [81].

## 5. Limitations of CAR-T-Cell Therapy in Multiple Myeloma

**Figure 3:**
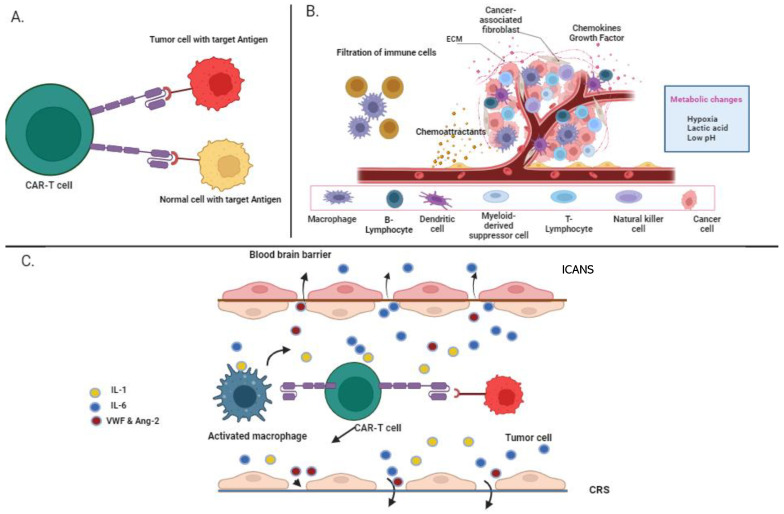
**Limitations of CAR-T-cell therapy:** (**A**) On-target/off-tumor attacking of the normal cell by CAR T cell if the same target antigen is present on the tumor cell. (**B**) The immunosuppressive microenvironment of the tumor cell consists of chemokines, and growth factors that lead to the infiltration of immune cells in the tumor. (**C**) CAR-T-cell toxicities: the pathogeneses of immune effector cell-associated neurotoxicity syndrome and cytokine release syndrome occur after the interaction of the tumor cell with the target antigen on the tumor cell.

The potential for CAR-T-cell technology is enormous, particularly in the treatment of hematological malignancies. As a result, many CAR-T products have been approved to treat a variety of hematologic malignancies. However, all these CAR-T-cell products have shown adverse side effects, such as neurotoxicity and CRS, in different clinical trials for MM (KarMMAa, CARTITUDE 1, LEGEND 2). Many structural limitations and their associated toxicities remain to be addressed (Figure 3).

### 5.1. On-Target, Off-Tumor

One of the most prevalent toxicities observed in CAR-T-cell therapy is the “on-target off-tumor” effect, whereby the same target antigen is expressed on normal cells, prompting the CAR T cells to inadvertently target healthy tissues, consequently inducing adverse effects. A notable reason for the development of numerous BCMA-based CAR constructs is the localized expression in mature MM plasma cells, a characteristic that is not uniformly observed with other targets, such as SLAMF7. The incorporation of a “suicide gene” into CAR-T constructs presents a potential solution, rendering modified CAR T cells subject to elimination in cases of toxicity [84]. The efficacy of this strategy hinges on the speed and thoroughness of the CAR-T-cell clearance, which might require several days, depending on the applied system.

An alternative approach involves the localized administration of CAR T cells to the disease site, which can mitigate the constraints of “on-target off-tumor” toxicity. This is possible due to the engagement of on-target activity solely within malignant tissue, thereby minimizing interaction with normal tissue. However, this approach is primarily applicable to solid cancers [85]. Another strategy is the design of co-inhibitory receptors (iCAR), a unique tactic that entails identifying surface antigens solely expressed by healthy tissue, distinct from tumor cells [86]. Furthermore, another approach incorporates the introduction of chemokine receptors to engineered CAR T cells, facilitating direct homing to the tumor microenvironment [87]. The prospective success of novel non-BCMA targets in MM critically hinges on the confined expression of these new targets in MM cells.

### 5.2. Cytokine Release Syndrome

Cytokine release syndrome represents systemic toxicity characterized by an upsurge in pro-inflammatory cytokines, such as IL-2, IL-6, IL-8, and IL-10. The CRS symptoms encompass a spectrum, from mild fever to potentially life-threatening capillary leak syndrome, hypotension, and even cardiac arrest, which can culminate in end-organ failure [88]. In the context of ALL, CRS occurrence correlates with CAR-T-cell activation, while in lymphomas, it aligns with the disease burden. A meta-analysis encompassing LEGEND-2 and KarMMa trials in RRMM demonstrated that around 80% of patients manifest CRS, out of which 14.1% experience grade 3 or higher toxicities [89]. The severity and frequency of CRS correlate with the dose of CAR T cells administered.

In CRS management, extended glucocorticoid administration (e.g., >14 days) is often employed, but this approach could compromise the functionality of infused CAR T cells [90]. Tocilizumab, an IL-6 receptor antagonist, gained FDA approval in 2017 for CRS treatment, displaying the rapid alleviation of CRS symptoms. Anti-IL-6 therapy is typically initiated at the onset of grade 2 CRS, with a substantial portion of patients requiring intensive care unit (ICU) support upon the emergence of grade 3 toxicities [89]. Yet, ongoing investigations are exploring the impact of IL-6 receptor inhibition on CAR-T-cell persistence and proliferation and antitumor effectiveness. Early indications suggest that the utilization of anti-cytokine agents does not curtail the antitumor potential of CAR T cells.

### 5.3. Immune Effector Cell-Associated Neurotoxicity Syndrome (ICANS)

Immune effector cell-associated neurotoxicity syndrome constitutes a neuropsychiatric disorder linked to immune cell activity. It emerges as a consequential complication after CAR-T-cell infusion. ICANS displays a variable clinical presentation, manifesting initially as toxic encephalopathy, dysphasia, and compromised motor function. In certain instances, additional symptoms such as motor weakness, seizures, and cerebral edema may arise [91]. Notably, a substantial proportion of patients experiencing ICANS have previously encountered and recovered from CRS, which could be perceived as an early indicator of ICANS.

Within the KarMMa study, neurological toxicities were identified in 45% of patients, with 6% of them having received glucocorticoid treatment [92]. In the CARTITUDE-1 trial, ICANS developed in 17% of patients [40]. The primary objective in managing ICANS is to mitigate the inflammatory response, with one approach involving the administration of an IL-6 antagonist. Siltuximab, serving as an IL-6 antagonist, impedes the continuous passage of IL-6 across the blood–brain barrier [93]. Steroids also demonstrate efficacy in ameliorating ICANS effects without compromising the efficacy of CAR T cells. As a preemptive measure for seizure prophylaxis, levetiracetam or alternative antiepileptic medications may be employed [94].

### 5.4. Immunosuppressive Microenvironment

In the tumor microenvironment, different cells contribute to immunosuppression, such as regulatory T cells (Tregs), tumor-associated macrophages (TAMs), and myeloid-derived suppressor cells (MDSCs) [95,96]. These tumor cells and infiltrates promote the production of chemokines, growth factors, and cytokines, which decrease antitumor activity due to immune checkpoint pathways like CTLA-4 and PD-1. The limited persistence period and poor T-cell expansion of CAR-T-cell therapy result in a weak response, suggesting that the co-inhibitory pathway mediates the development of T-cell exhaustion [97]. The integration of CAR T cells and checkpoint blockades in combination with immunotherapy is regarded as the next significant advancement in this field, as CAR T cells provide infiltration and PD-1/PD-L1 [98]. Recently, the focus has been on engineering CAR T cells that are resistant to tumor microenvironment immunosuppressive factors, like TGFβ-mediated inhibitory signals [99]. Another promising method involves engineering CAR T cells by adding immunostimulatory signals and producing stimulatory cytokines that promote T-cell survival and expansion and antitumor activity while re-equalizing the tumor microenvironment [27]. Therefore, more research is required to determine how checkpoint blockage and CAR-T-cell therapy interact with various immunotherapies to affect T-cell infiltration and effector-function indifferent malignancies.

## 6. Recent Advances in BCMA Bispecific Antibodies

Recent advancements in multiple myeloma (MM) therapy have been notably cantered around treatments aimed at the B-cell maturation antigen (BCMA) [100]. While CAR-T-cell therapy for the BCMA has been a prominent focus, BCMA bispecific antibodies have emerged as a promising alternative for addressing relapsed and refractory MM (RRMM). These therapeutic agents have garnered attention due to their encouraging performance in clinical trials, providing a substantial addition to the continually evolving landscape of MM treatments. For instance, belantamab mafodotin, a bispecific antibody targeting the BCMA on malignant plasma cells and delivering the potent microtubule inhibitor monomethyl auristatin F, has received approval from the US Food and Drug Administration (FDA). Furthermore, teclistamab, another anti-BCMA bispecific antibody, is in the advanced stages of gaining commercial approval. In essence, BCMA bispecific antibodies have demonstrated promising clinical trial results and are poised to play a pivotal role in the treatment of RRMM [100,101].

### 6.1. Comparison with BCMA CAR-T Therapy

BCMA bispecific antibodies and BCMA CAR-T-cell therapy share the common goal of targeting BCMA-expressing myeloma cells, albeit through distinct mechanisms with unique characteristics. Unlike CAR-T-cell therapy, BCMA bispecific antibodies are readily available as “off-the-shelf” products, eliminating the necessity for personalized, patient-specific cell manufacturing. This characteristic not only streamlines the treatment process but also mitigates some of the logistical challenges associated with CAR-T therapy. Moreover, BCMA bispecific antibodies typically present a distinct safety profile, characterized by a reduced risk of severe cytokine release syndrome (CRS) and immune effector cell-associated neurotoxicity syndrome (ICANS) when compared to CAR-T therapy. These attributes render BCMA bispecific antibodies particularly appealing for patients with specific comorbidities or higher susceptibilities to severe adverse events.

### 6.2. Potential Synergies and Complementary Roles

To address the multifaceted challenges entwined with RRMM treatment, exploring the potential synergies and complementary roles of BCMA bispecific antibodies and BCMA CAR-T therapy is crucial. Several strategies warrant consideration, such as the adoption of sequential therapy. Here, BCMA bispecific antibodies can be administered initially to curtail the tumor burden and manage the disease progression swiftly. Subsequently, BCMA CAR T cells can be introduced to establish a more enduring and resilient response, potentially mitigating concerns surrounding antigenic escape and ensuring protracted disease control. Furthermore, the concurrent or sequential utilization of BCMA bispecific antibodies and CAR-T therapy holds significant promise and deserves further scrutiny, offering tailored approaches aligned with the patient’s clinical status and individual requirements.

The incorporation of BCMA bispecific antibodies into the armamentarium of MM treatment charts is a promising course to address the unmet medical needs of RRMM patients. By comparing the effectiveness, safety, and practical considerations of BCMA bispecific antibodies with BCMA CAR-T therapy and delving into their potential synergies, a more dynamic and adaptable future for MM therapy comes into view. As ongoing clinical trials and real-world data continue to accrue, the strategic deployment and integration of BCMA bispecific antibodies and BCMA CAR-T therapy are poised to play a pivotal role in propelling RRMM management forward. Ultimately, these innovative therapies propel us closer to the aspiration of achieving sustained remission and enhancing the quality of life for MM patients.

## 7. Conclusions

Immune-based therapies, particularly CAR-T-cell therapy, have shown promising results in treating RRMM. In this review, we provide an overview of CAR T cells’ scientific and technical aspects and discuss recent clinical studies that target MM using a variety of antigen targets, of which BCMA is the most promising. Early-phase trials of BCMA CAR-T-cell therapy show promise, but several challenges remain, such as toxicity and poor responses in some patients. New strategies are being developed to overcome these shortcomings. The introduction of dual-targeting CARs or suicide genes may result in the better efficacy of CARs and the avoidance of antigen escape. With combined efforts between industry partners and academic institutions, CAR T cells have the potential to offer definitive therapy options for MM.

## Figures and Tables

**Table 1:** Clinical Studies in Multiple Myeloma.

**(A) BCMA Targeted CAR-T for MM**					
**Clinical Trial Name**	**Antigen Target**	**CAR Name**	**Sponsor**	**Phase**	**Current Status**
NCT05393804	BCMA	Idecabtagene Vicleucel (ide-cel, bb2121)	Memorial Sloan Kettering Cancer Cente	2	Recruiting
NCT05347485	BCMA	Ciltacabtagene Autoleucel	Janssen Scientific Affairs, LLC	2	Recruiting
NCT04181827	BCMA	JCARH125	Janseen Research & Development	3	Active/Non-recruiting
NCT03090659	BCMA	LEGEND-2	Nanjing Legend Biotech Co. Second Affiliated Hospital of Xi’an Jiaotong University Ruijin Hospital Jiangsu Provincial People’s Hospital Shanghai Changzheng Hospital	1/2	Active, not recruiting
NCT03548207	BCMA	CARTITUDE-1	Janssen Research & Development, LLC	1/2	Completed
NCT02954445	BCMA	NA	Shiqi Li, Southwest Hospital, China (Responsible Party) Southwest Hospital, China	1/2	Unknown
NCT05860036	BCMA	NA	Institute of Hematology & Blood Diseases Hospital Gang An, Institute of Hematology & Blood Diseases Hospital (Responsible Party)	1	Recruiting
NCT05740891	BCMA	NA	Zhejiang University He Huang, Zhejiang University	1	Recruiting
NCT03716856	BCMA	NA	First Affiliated Hospital of Zhejiang University	1	Unknown
NCT05594797	BCMA	NA	Hrain Biotechnology Co., Ltd.	2	Recruiting
NCT04003168	BCMA	NA	Hrain Biotechnology Co., Ltd.	1	Recruiting
NCT03338972	BCMA	NA	Fred Hutchinson Cancer Center	1	Completed
NCT04727008	BCMA	CXCR4	Sichuan University Ting Niu, West China Hospital	Early Phase 1	Not Yet Recruiting
NCT05698303	BCMA	NA	Nanjing IASO Biotherapeutics Co., Ltd.	1	Not Yet Recruiting
NCT04637269			Xinqiao Hospital of Chongqing Xi Zhang, MD, Xinqiao Hospital of Chongqing	Early Phase 1	Recruiting
NCT04322292	BCMA	C-CAR088	Institute of Hematology & Blood Diseases Hospital AnGang, Institute of Hematology & Blood Diseases Hospital	1	Unknown
**(B) Non-BCMA Targeted CAR-T for MM**					
**Clinical Trial Name**	**Antigen Target**	**CAR Name**	**Sponsor**	**Phase**	**Current Status**
NCT04674813	GPRC5D	CC-95266	Juno Therapeutics, a Subsidiary of Celgene	1	Active, Recruiting
NCT05016778	GPRC5D	POLARIS	Zhejiang University & OriCell Therapeutics Co., Ltd.	Early Phase 1	Active, not recruiting
NCT05431608	GPRC5D	NA	Memorial Sloan Kettering Cancer Center	1	Active, Recruiting
NCT05325801	GPRC5D & BCMA	OriC321	Zhejiang University	1	Recruiting
NCT05509530	GPRC5D & BCMA	NA	Xuzhou Medical University	2	Recruiting
NCT03958656	SLAMF7	NA	National Cancer Institute (NCI)	1	Completed
NCT03710421	CS1	NA	City of Hope Medical Center	1	Active, Recruiting
NCT04499339	SLAMF7	CARAMBA-1	European Commission and Wuerzburg University Hospital	1/2	Active, Recruiting
NCT04142619	SLAMF7	UCARTCS1	Cellectis S.A	1	Active, Recruiting
NCT03464916	CD38	CAR2 Anti-CD38 A2	Sorrento Therapeutics, Inc.	1	Completed
NCT03767751	CD38	NA	Chinese PLA General Hospital	1/2	Unknown
NCT02135406	CD19	CTL019/tisagenlecleucel	University of Pennsylvania	1	Completed
NCT03672318	CD138	ATLCAR.CD138	UNC Lineberger Comprehensive Cancer Center	1	Active, Recruiting
NCT01886976	CD138	CART-138	Chinese PLA General Hospital	1/2	Unknown
NCT04182581	BCMA/CD19	BCMA/CD19 Dual-Target	Xijing Hospital	Early Phase1	Unknown
NCT03706547	CD19	NA	Peng Liu Peng Liu, Shanghai Zhongshan Hospital	1	Unknown
NCT04714827	CD19	CD19-CD22	Shanxi Province Cancer Hospital	1/2	Recruiting
NCT04541368	SLAMF7	CS1	Zhejiang University He Huang, Zhejiang University	Early Phase 1	Not Yet Recruiting

## Data Availability

Not applicable.
